# Barriers and strategies in care delivery for type 1 diabetes in Sub-Saharan Africa: a scoping review

**DOI:** 10.1186/s12889-026-27354-9

**Published:** 2026-05-06

**Authors:** Hervé Brice Djiofack Kentsop, Christina Zarowsky, Julia Elisabeth von Oettingen

**Affiliations:** 1 Département de médecine sociale et préventive, École de santé publique, Université de Montréal, 7101 Park Ave, Montréal, Québec H3N 1X9 Canada; 2 Centre de recherche en santé publique, Université de Montréal et CIUSSS du Centre-Sud-de-l’Île-de-Montréal, Montréal, Canada; 3 Research Institute, McGill University Health Centre, Montreal, Québec H4A 3JI Canada; 4 Department of Pediatrics, Division of Pediatric Endocrinology, McGill University Health Centre, Montreal, Canada

**Keywords:** Care delivery, Type 1 diabetes, Scoping review, Obstacle, Strategy, Sub-Saharan Africa

## Abstract

**Background:**

Type 1 diabetes is one of the most common chronic diseases in children. More than 1.2 million young people under the age of 20 are affected, the majority of whom live in low-income countries. The care delivery of type 1 diabetes in sub-Saharan Africa is an important part of protecting children’s health, preventing complications, and supporting families in vulnerable populations. This scoping review aims to describe and categorize the most important barriers to type 1 diabetes care delivery in Sub-Saharan Africa and provides an overview of the types of strategies that are used to overcome these barriers.

**Methods:**

Scoping review methodology was used. Five online databases and a general web search for grey literature were searched. Eligibility criteria were applied by two reviewers, and data were extracted and analysed by one reviewer. All studies published or unpublished from 1990 until the end of 2020 were considered. The content was analyzed using a qualitative synthesis approach.

**Results:**

Research, including publications from 1990 to 2019, identified 504 studies, narrowed down to 38 after review. All studies were based in sixteen Sub-Saharan African countries, limiting the generalizability of our results. Barriers to the care delivery of type 1 diabetes in Sub-Saharan Africa are related to contextual factors at several levels: health system, policy, structure and performance, patient factors, and socio-cultural factors. The core elements of successful strategies for care delivery of type 1 diabetes are education, staff training, and social support.

**Conclusions:**

Available evidence indicates that structured care delivery can improve the health of people living with type 1 diabetes in Sub-Saharan Africa. Obstacles in the case and population care delivery of type 1 diabetes and the health of people living with type 1 diabetes need to be analyzed before strategies adapted to the context and specific target groups can be developed.

**Supplementary Information:**

The online version contains supplementary material available at 10.1186/s12889-026-27354-9.

## Background

Type 1 diabetes (T1D) is a serious chronic condition associated with a risk of life-threatening complications, and the financial burden associated with managing this disease is high. More than 1.2 million young people under the age of 20 have T1D, and more than half of them live in low-middle income countries [1]. T1D exists in Sub-Saharan Africa (SSA), but its incidence is underestimated [2], and estimates vary widely across countries. Recent studies have reported an annual incidence of 10.1/100,000 in Kano among children under 15 years of age [3]. The peak age of onset al.so varies widely: 15–19 years in Tanzania, 22–23 in South Africa, and 20–25 in Ethiopia [4].

Today, T1D in children is a particularly challenging problem in Africa [5, 6]. At the patient and family level, managing T1D includes constant surveillance of blood glucose at a minimum of 4–10 times per day; insulin injections at a minimum of 3 times per day; nutritional management, including carbohydrate counting; treatment for hypo- and hyperglycemia; knowledge of all factors influencing blood sugar levels and interpretation of blood glucose trends and adjustment of treatment regimens. Phenotypes of T1D in this region are poorly characterized, and information regarding etiology and epidemiology is scarce [7]. In SSA, the lack of financial resources and education, food insecurity, difficulty accessing health care as well as insulin and care supplies, and stigma make the care delivery of T1D even more difficult [8, 9]. Care delivery interventions at both the population and clinical care levels should include multisectoral approaches to enable country infrastructures to support workflow-focused or provider-focused T1D care [10].

The specific research question for this review is as follows: What are the obstacles and strategies put in place in the care delivery of T1D in SSA from 1990 to 2020? The first objective of this scoping review was to identify the obstacles to care delivery of T1D in ASS. The second objective was to identify the strategies implemented to integrate actions for effective management. Overall, this review was to describe and categorize the most important obstacles and report effective strategies implemented in the care delivery of T1D patients in SSA.

## Methods

### Study design

This scoping review follows the methodology proposed by Arksey and O’Malley [11] which included identifying relevant sources of evidence, selecting sources of evidence, charting the data, and summarizing the results. The review is reported following the Preferred Reporting Items for Systematic reviews and Meta-Analyses extension for Scoping Reviews (PRISMA-ScR) (Additional file 1) [12]. This protocol is registered with the Open Science Framework, and ethics approval was not required as the review relied solely on publicly available information. We describe the review process using these four steps, which were carried out by DKHB and overseen by CZ and JVO through regular meetings and discussion.

### Identifying relevant sources of evidence

First, the search strategy was conducted in MEDLINE (PubMed) and CINAHL (Cumulative Index to Nursing and Allied Health Literature) to identify articles on the analyzed topic. The text words contained in the titles and abstracts of the articles considered relevant were consulted and the index terms were used to develop a comprehensive search strategy. Then, with the help of a research librarian (SF) from the University of Montreal, an initial search was conducted to develop and refine the scientific literature search strategy. The searches were conducted in collaboration with the research librarian using the following databases: Embase, Global Health, PubMed, Science direct, Web of Science. The searches used both controlled vocabularies, such as Medical Subject Headings (MeSH), and keywords representing concepts such as: (diabetes or type 1 diabetes or diabetes mellitus) AND (management or care delivery) AND (programs) AND (Sub-Saharan Africa). The scientific literature search focused on articles published between 1990 and 2020. A limiter related to the publication date has been applied. The MEDLINE search strategy is available in Additional File 2. The following search algorithm was used:

[“management” or “care and support” or “taking care of” or “support”] and [“Diabet* ” or “ T1DM ” or “T1D” or “ diabetes mellitus ”] and [“africa” or “west* africa*” or “east* africa*” or “centr* africa*” or “south* africa*” or “Angola” or “Burundi” or “Democratic Republic of Congo” or “DRC” or “Cameroon” or” Central African Republic” or “CAR” or “Chad” or “Republic of Congo” or “Equatorial Guinea” or “Gabon” or “Kenya” or “Nigeria” or “Rwanda” or “Sao Tome and Principe” or “Tanzania” or “Uganda” or “Sudan” or “South Sudan” or “Djibouti” or “Eritrea” or “Somalia” or “Botswana” or “Comoros” or “Lesotho” or “Madagascar” or “Malawi” or “Mauritius” or “ Mozambique” or “Namibia” or “Seychelles” or “South Africa” or “Swaziland” or “Zambia” or “Zimbabwe” or “Benin” or “Mali” or “Burkina Faso” or “Cape Verde” or “Ivory Coast” or “Gambia” or “Ghana” or“Guinea” or “Guinea Bissau” or “Liberia” or “Mauritania” or “Niger” or “Senegal” or “Sierra Leone” or “Togo”.tw,* kw].*

Finally, the reference lists of studies included in this review were searched, but no additional relevant studies were found.

### Selecting sources of evidence

All studies identified in the search were collected and entered EndNote (version X9.3.3), and duplicates were removed. This review included a wide range of published and unpublished studies encompassing meta-analyses, randomized controlled trials (RCTs), case-control studies, case series and case studies that assessed care delivery strategies of T1D and the factors associated with T1D in SSA in a young diabetic population. Theoretical conceptual documents obtained from Google Scholar were also included to identify the concepts relating to the theme to broaden our field of research. A filter restricted the search to studies and reviews limited to children aged 0–18, in French and English languages. All articles indexed in the 5 databases and published from 1990 until the end of 2020 were considered. (1990 - date on which most of the care delivery strategies related to T1D in SSA seem to have emerged following the Bamako initiative (primary health care rehabilitation program launched in 1987 by James GRANT, director of the United Nations Children’s Fund -UNICEF) and the constraints encountered in the implementation of primary health care and the ambition of health for all).

Titles and abstracts were screened by two independent reviewers to assess the previously defined inclusion criteria (Table 1). Disagreements between reviewers were resolved through constructive discussion and consensus was reached without the need for a third reviewer. After assessing all titles and abstracts, studies that met the inclusion criteria were read in full. We used the following inclusion criteria for selecting titles and abstracts: studies with empirical data on care delivery of patients with T1D; studies based in an SSA country. Reviews were excluded, but no reviews of diabetes-related experiences were identified. If abstracts indicated that they focused on care delivery of other groups of people, such as patients with type 2 diabetes (T2D), healthcare professionals, or caregivers, they were also excluded. Once the titles were screened, the same inclusion and exclusion criteria were applied to the abstracts.

**Table 1:** Eligibility criteria

Inclusion criteria	Exclusion criteria
Reviews, commentaries and documents on both T1D and T2D	Studies exclusively on T2D and its complications
Data on the management of T1D	The results of T1D in studies that did not use only the young diabetic population
The diabetic population in Sub-Saharan Africa	
Data from the period between 1990 and 2020	
Original research, World Health Organization research, theses and reviews published in French and English	

### Charting the data and summarizing the results

The charting form was pilot tested by two reviewers with a random sample of 5 sources of evidence from the scientific or grey literature to ensure that all relevant data were captured. Eligible sources of evidence were recorded independently by one reviewer. We also looked for grey literature, guidelines, reports from important agencies including the International Diabetes Federation, the World Health Organization and International Society for Pediatric and Adolescent Diabetes. Only data relevant to care delivery of T1D in SSA were recorded (i.e. information on care delivery of T2D or results that were not based on a young population were not recorded), in line with the a priori objectives of the review. The extracted data were presented in a table format and a narrative synthesis accompanies the results, describing how the findings relate to the objective and question formulated for this review. The content of the articles was analyzed using a qualitative synthesis approach [13] which made it possible to bring together various perspectives in terms of theoretical bases, techniques for collecting and analyzing data and contextual information, to identify important obstacles and strategies in the care delivery of T1D in SSA. During the analysis process, particular attention was paid to the link between obstacles and adequate strategies in time and space. Studies and reviews were included in the synthesis. If obstacles and/or strategies were mentioned, they were included in the reference framework that we developed for our study.

## Results

**Fig. 1:**
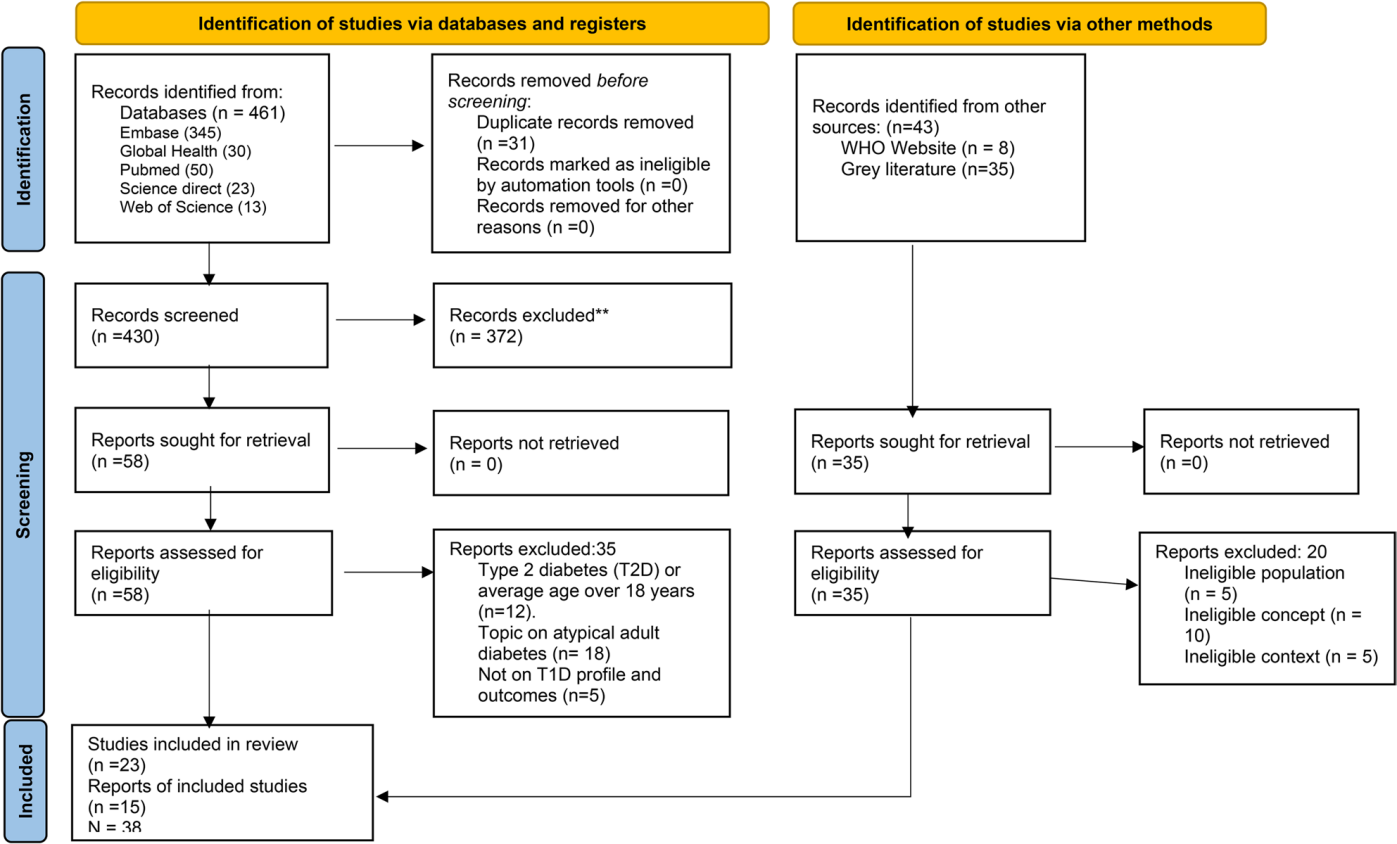
PRISMA-ScR flowchart for our scoping review

Our PRISMA-ScR flowchart is shown in Fig. 1. The search algorithm developed above identified 504 articles including gray literature and organizations’ websites. After reviewing the title and abstract and identifying duplicate articles, 430 of 504 articles were excluded. 73 articles were considered in the selection of full texts. An additional 35 were excluded for the following reasons: T2D or average age over 18 (*n* = 12); topic on atypical adult diabetes (*n* = 18); no direct reference to results in relation to obstacles and evolution of T1D care delivery in SSA (*n* = 15). Overall, the 38 articles included in the review consisted of 16 empirical studies and 22 theoretical reviews or concept papers (Table 2). All the studies focused on disease care delivery strategies in relation to obstacles in SSA. The geographic scope included several unspecified countries (*n* = 16), grouped countries (*n* = 2), Cameroon (*n* = 4), South Africa, Tanzania, Sudan, and Ethiopia respectively (*n* = 2) and Uganda, Ghana, Mali, Benin, Rwanda, Gabon, Kenya, and Nigeria.

**Table 2:** Studies included in the qualitative synthesis

Author(s), year, country	Aims	Type of study	Results
Atun R. et al., 2017, 12 SSA countries [14]	To determine the current burden of diabetes, its risk factors and outcomes in the region, to assess the challenges faced by health systems in addressing this burden and to suggest potential solutions	Literature review	At present, many of the health systems in sub-Saharan Africa struggle to cope with infectious diseases. Meeting the goals of the UN high-level meeting on NCDs (to reduce premature mortality from NCDs by 25% by 2025) and Sustainable Development Goals (SDGs; to reduce premature mortality from NCDs by a third by 2030) requires a coordinated approach within countries, which starts with a firm consideration of disease burden, needs, and priorities
Bahendeka SK, 2016, Uganda [10]	Advocating for improved T1D care in sub-Saharan Africa.	Cross-sectional study	Many participants identified with hyperglycaemia (90.5% IFG and 48.9% diabetes) were not aware of their hyperglycaemic status. Factors associated with IFG were region of residence, body mass index and total cholesterol; factors associated with diabetes mellitus were age, sex, household floor finish and abdominal obesity.
Beran et al., 2006, Mozambique, Zambia [15]	To assess barriers to the management of patients with insulin-dependent diabetes in Mozambique and Zambia.	Case study	In each country the protocol was implemented in 3 areas – the capital city, a large urban centre and a predominantly rural area and their respective surroundings. Interviews were carried out by local teams trained on how to use the tool. Data was then collected and entered a database for analysis.
Bigna et al., 2018, Cameroon [16]	To summarize current data on the prevalence of prediabetes and diabetes mellitus in Cameroon.	Cross-sectional studies	All included studies had a low risk of bias. Six studies were conducted in an urban setting only, one in a rural setting only, and five in both settings. In univariable meta-regression analysis, the prevalence of diabetes mellitus increased with age, hypertension, overweight and obesity. There was no difference for sex and settings (rural versus urban)
Borus et al., 2020, Not indicated [17]	Review barriers to adherence and discuss promising interventions to improve child outcomes	Synthesis review	Effective interventions build on teens’ internal and external supports (family, technology, and internal motivation) to simplify their management of diabetes and provide opportunities for the teens to share the burdens of care.
Butler et al., 2008, Not indicated [18]	To identify modifiable family factors impacting glycemic control in youth with type 1 diabetes (T1DM) beyond the anticipated physical, developmental, and behavioral issues associated with adolescence.	Cohort study	Higher parental knowledge, less youth negative affect, and less parental burden predicted lower HbA1c, while youth knowledge and parental negative affect did not.
Datye et al., 2015, Not indicated [19]	To examine barriers to adherence among adolescents with T1D and discuss interventions to improve treatment adherence and glycemic control	Systematic review	Interventions include technology-based applications, family-based therapies, motivational interviewing, and others. Notably, less than 10% of the interventions reviewed are provider-led, clinic-based interventions, and few have focused on regimen-related aspects of adherence.
Djonou Cathy et al., 2019, Not indicated [20]	To describe the prevalence of glycemic control and related factors in a population of patients with T1D in sub-Saharan Africa.	Cross-sectional study	We enrolled 95 children adolescents, aged from 06 to 19 years. There was an association between study level of the patients, healthy eating habits, diabetes duration and level of glycemic control on univariate analysis. On multivariate analysis, diabetes diagnosed for more than 2 years was associated to a good control compared to those with diagnosis that is more recent. Glycemic control of adolescents with type1 diabetes remain very poor in Cameroon despite the implementation of free diabetes care through the program CDiC.
Fagbemi et al., 2017, Benin, [21]	To determine the sociodemographic characteristics, assess the frequency of type 1 diabetes (T1D) and calculate the mortality rate among type 1 diabetics who consulted the Cotonou Insulin Bank from January 1996 to December 2015	Retrospective study	In total, 12,000 files were studied, including 103 files of T1D patients. The frequency of T1D was found to be 0.9%. A mortality rate of 34% was noted in the group of type 1 diabetic patients with a mortality rate of 54.3% in men and 45.7% in women.
Fedaku et al., 2010, Ethiopia, [22]	Assess social and nutritional determinants.	Case-control study	Diabetes was strongly associated with subsistence farming and illiteracy/low levels of education. Diabetes was also linked with a history of childhood malnutrition, the mother’s death during childhood and markers of poverty including poorer access to sanitation, greater overcrowding, increased distance from the clinic and having fewer possessions. In addition, men with the disease tended to be shorter, were lighter with reduced sitting height and reduced biacromial and bitrochanteric diameters.
Gill et al., 2005, South Africa, [23]	To assess long-term (20-year) mortality, with causes of death, in a cohort of type 1 diabetic patients residing in Soweto, South Africa.	Cohort study	Of the original cohort of 88 Type 1 patients, 21 died during the follow-up period. There were 39 lost to follow‐up, giving a crude 20 years’ mortality of 43%. Other causes were hypoglycaemia, ketoacidosis, infection and undetermined. Of the survivors, comparing data at 0- and 20-years’ follow‐up, there was a significant increase in rates of retinopathy and hypertension, but not of other complications.
Hall et al., 2011, Not indicated, [6]	To provide a comprehensive and up-to-date review of epidemiological trends and public health consequences of diabetes in sub-Saharan Africa	Systematic Review	Diabetes is likely to increase the risk of several important infections in the region, including tuberculosis, pneumonia and sepsis. Meanwhile, antiviral treatment for HIV increases the risk of obesity and insulin resistance. Barriers to accessing diagnosis and treatment included a lack of diagnostic tools and glucose monitoring equipment and high cost of diabetes treatment.
Jaffiol et al., 2011, Not indicated, [9]	Show that care comes up against multiple difficulties relating to the organisation of the health system, the limitation of health budgets largely absorbed by the fight against communicable diseases, the lack of patient education and the impossibility for many to obtain insulin and antidiabetic drugs, and finally cultural habits	Systematic review	The care is faced with multiple difficulties due to the organization of the health care system that is unable to cope with the epidemic, the limitation of health budgets largely absorbed by the fight against communicable diseases, the lack of patient education and the impossibility for many to obtain insulin and antidiabetic drugs, and finally due to cultural habits that make it difficult to accept hygiene and dietary messages. The fight against the disease requires its prevention as a priority, which is essential to reduce its incidence.
Kratzer et al., 2012, Ghana, [24]	To explore the structural barriers faced by families with type 1 diabetes mellitus in Ghana and provide insights for policy development	Qualitative study	Participants expressed concern over the misdiagnosis of T1DM at primary care facilities, resulting in some of the children going into a diabetic coma before receiving proper care. Children and parents noted discrimination and poor care at school. Financial burden was due to the high costs of medications and appliances needed for proper diabetes management. A lack of formal support was credited by participants to be the result of the lethargy of advocacy groups or resource centers. Finally, there was a lack of readily available and accessible information for children and parents on T1DM
Kim et al., 2012, Not indicated, [25]	To determine the relationship between duration of persistent glycemic control in children with type 1 diabetes and the likelihood of subsequent improvement.	Retrospective cohort study	Patients aged 12–18 years, females, and Medicaid patients were twice as likely to be in persistently poor control as patients aged 6–11 years, males, and privately insured patients, respectively. Of the 39 patients with improved control, only 5 sustained their improvement for ≥ 2 years. Multiple contributing factors for improved control were identified, but no one factor explained improved control in > 25% of patients.
Koki et al., 2013, Cameroon, [26]	Determine the profile of ophthalmological pathologies maintained by glycemic imbalance in children and adolescents with type 1 diabetes	Prospective cross-sectional study	Infectious oculo-palpebral pathologies, here maintained by glycemic imbalance, are multiple and varied. The severity depends on the duration and, above all, on the glycemic balance, which is difficult for the child to maintain. The prevention of these pathologies would therefore involve increased awareness of the systematization of quarterly HbA1c testing of diabetic children by caregivers, involvement of both parents, and good individual and environmental hygiene of those being cared for, to preserve their visual future.
Marshall et al., 2015, Rwanda, [27]	To determine estimates of the prevalence and incidence of clinically recognized cases of type 1 diabetes	Retrospective study	The prevalence of known T1D in seven districts in Rwanda for ages < 26 years was 16.4/100 000 and for < 15 years was 4.8/100 000. Prevalence was higher in females than males and rates increased with age. The annual incidence rate for those < 26 years was stable between 2007 and 2011 with a mean incidence over that time of 2.7 /100 000. Incidence rates were higher in females than males and peaked in males at ages 17 and 22 years and in females at age 18 years
Mawo et al., 2015, Cameroon, [28]	To determine the frequency and type of dermatological conditions found on physical examination of diabetic patients	Cross-sectional and descriptive study	Among 231 patients enrolled, a total of 22 types of dermatological lesions were observed, with 44.1% of patients presenting at least one dermatological condition. These conditions were classified in three categories: lesions associated with diabetes, lesions related to diabetes complications, and lesions related to diabetes treatment. Dermatological conditions related to diabetes complications were the most frequently observed with a predominance of fungal infections in 66.3% of affected patients.
Mbanya et al., 2010, Not indicated, [29]	Not indicated	bibliographic research	The rate of undiagnosed diabetes is high in most countries of sub-Saharan Africa, and individuals who are unaware they have the disorder are at very high risk of chronic complications.
Notkins et al., 2001, Not indicated, [30]	Summarize some generally accepted facts about this disease	Scoping review	The studies on autoimmunity have provided clinically useful information. In particular, the demonstration of the presence of autoantibodies years before the onset of clinical symptoms has made it possible to identify individuals at high risk of developing type 1 diabetes and to initiate therapeutic intervention trials on relatively small numbers of subjects.
Padoa, 2011, South Africa, [7]	Overview of the epidemiological, immunological and genetic data that have been generated on T1D in Africa, particularly in the black population	Literature review	The prevalence of type 1diabetes is thought to be lower in Africans than Europeans. Therefore, there may also be significant ethnic differences in both the aetiology and pathology of the disease
Piloya-Were et al., 2016, Not indicated [31]	Summarizes the current state of diabetes in African children.	Literature review	There is still a long way to go before the standard-of-care available to children in resource-rich nations is available to children with diabetes in Africa
Quintal et al., 2019, Not indicated [32]	Identify and articulate ethical issues related to the use of the artificial pancreas for patients, health professionals, the private sector and policy makers	Literature review	We identified five sensitive domains of ethical issues. Patient confidentiality and safety can be jeopardized by the artificial pancreas’ vulnerability to security breaches or unauthorized data sharing. Public and private coverage of the artificial pancreas could be cost-effective and warranted. Patient selection criteria need to ensure equitable access and sensitivity to patient-reported outcomes. Patient coaching and support by healthcare professionals or industry representatives could help foster realistic expectations in patients. Finally, the artificial pancreas increases the visibility of diabetes and could generate issues related to personal identity and patient agency
Sap et al., 2019, Cameroon, [33]	To evaluate the short-term impact of patient education via WhatsApp on disease knowledge and glycemic control in adolescents and young adults living with T1D in a resource-limited setting	A double arm non-randomized clinical trial	We recruited 54 patients of which 25 subjects and 29 controls. Median age was 19 and 19 years for the intervention and control group, respectively. There was a significant improvement of knowledge on diabetes in the intervention group from 13/20 to 16/20 after 2 months. The mean proportion of acute complications decreased from 28% to 16% in the intervention group and increased from 7% to 34% in the control group. There was no improvement in glycosylated hemoglobin level in both groups.
Sidibé et al., 1999, Mali [34]	Show how mobilizing field actors, with the support of non-governmental organizations (NGOs), can improve and enhance the care of diabetic patients	Observational prospective study	-
Silverstein et al., 2005, Not indicated [35]	Provide a single resource on current standards of care pertaining specifically to children and adolescents with type 1 diabetes	Systematic review	Children have characteristics and needs that dictate different standards of care. The management of diabetes in children must take the major differences between children of various ages and adults into account.
Swai et al., 1993, Tanzania, [36]	To ascertain the annual incidence of diabetes requiring treatment with insulin in children and adolescents aged 0–19 years in Dar es Salaam, Tanzania, during a 10-year period from 1 January 1982 to 31 December 1991.	Prospective registration study	The annual incidence of juvenile diabetes for both sexes was 1.5 per 100,000 population aged 0–19 years. Incidence per 100,000 population per year increased with age: 0.6 in the age group 0–4 years, 0.5 at 5–9 years, 2.2 at 10–14 years, and 3.4 at 15–19 years.
Takogue et al., 2014, Cameroun [4]	The aim of this study was to assess the evolution of the average preprandial blood sugar levels and insulin requirements in diabetic children and adolescents participating in a camp organized in Yaoundé in August 2012.	Prospective registration study	Twenty-seven patients participated, 12 G/15 F. The mean duration of diabetes was 2.8 ± 2.8 years. Between the first and last day, no significant differences were observed in the mean insulin dose and mean preprandial blood glucose levels. Most children had at least one episode of hypoglycemia (74%). Most hypoglycemias (54%) occurred between 7:00 p.m. and 10:00 p.m. compared to only 8% between 7:00 a.m. and 10:00 a.m.
Angamo et al., 2013, Ethiopia [37]	The objective of this study is to identify determinants of glycemic control among insulin treated diabetic patients at Jimma University Hospital, Southwest Ethiopia.	Cross-sectional study	Patients had a mean age of 41.37 years, 58.5% were males, the mean duration of insulin treatment was 4.9 years, 18.3% achieved good glycemic control, 95% self-reported repeated use of disposable insulin syringe-needle and 48% correctly rotating insulin injection sites. Most of study participants had one or more complications. On multivariable logistic regression analyses, body weight of > 70 Kg, total daily dose of insulin ≤ 35 IU/day, total daily dose variation without checking glycemic level, knowledge deficit about signs and symptoms of hyperglycemia, and non-adherence to dietary management were independent predictors of poor glycemic control.
Damiens et al., 2019, Gabon [38]	Describe epidemiological aspects of type 1 diabetes in children in Gabon, specify the difficulties met by the patients during the follow-up and identify factors explaining poor therapeutic observance and metabolic control	Prospective transversal monocentric study	306 patients (154 girls and 152 boys) were diagnosed with T1D during the study period and followed-up in the Endocrinology Department at CHUL. hospital mortality was about 7%. Number of factors linked with poor therapeutic observance (high cost of treatment, lack of patients’ therapeutic education, etc.) were identified.
Ngwiri et al., 2015, Kenya [39]	Determine the degree of control in patients with T1DM aged 1–19 years over a 6-month period in 3 outpatient Kenyan clinics. It also sought to determine how control was influenced by parameters of patient and treatment.	Quantitative study	The median HbA1c for the study population was 11.1% (range: 6.3–18.8). Overall, only 28% of patients had reasonable glycemic control as defined in this study. 72% therefore had poor control. It was also found that age above 12 years was significantly associated with poor control.
Majaliwa et al., 2008, Not indicated [40]	Put together the published data on T1DM in African children, to document the burden of the disease, to identify major challenges in its management in the sub region, and to offer solutions	Literature review	An effective management and/or prevention of diabetes and its complications in Sub-Saharan African children should adopt multidisciplinary approaches. To improve the treatment of diabetes patients in developing countries, specialized clinics need to be established
Umar, 2016, Nigeria [41]	To examine the prevalence of Type 1 diabetes in children at the Aminu Kano Teaching Hospital, Kano.	Retrospective study	A total of 7929 patients were seen during the study period, out of which 18 were diagnosed with T1DM, giving a case prevalence rate of 2.3/1000. Sixteen of the 18 patients were first presented with DKA. The mean age at presentation was 8 years, and there were more females 13/18 than males 5/18 among the subjects. The most prevalent presenting symptoms were dehydration 16, fever 14, abdominal pain 12, polyuria and polydipsia 12. Two of the sixteen patients with DKA died with cerebral oedema during admission.
Elrayah et al., 2005, Sudan [42]	To estimate the direct costs of childhood diabetes in a low-income country, Sudan, and to assess the effectiveness of care paid for by the families	Descriptive cross-sectional study	The low direct costs reflect the minimal care given to the diabetic patients. Under the present economic conditions, families pay a considerable part of their income to sponsor the health of their diabetic children and receive little support other than that from relatives and friends. The present organization of diabetes care does not provide the patient with empowerment, knowledge and self-care ability. Well-trained diabetic teams and education programs may improve this situation.
Makame, 1992, Not indicated [43]	Not indicated	Systematic review	Two major issues of importance related to Type 1 diabetes in African and other developing countries are missed diagnosis and unavailability of insulin, issues which cannot be ignored
Motala et al., 2003, Not indicated [44]	Not indicated	Study report	For type 1 diabetes, the limited available data suggest that in SSA, the frequency is low, and that age of onset occurs later than in the western world. There is evidence for the role of genetic and immunological factors in its pathogenesis
Beran et Yudkin, 2006, Not indicated [45]	To review current evidence on diabetes care in sub-Saharan Africa and propose an 11-point action plan to address this problem in the region	Systematic review	-
Majaliwa, 2007, Tanzania [46]	To assess glycemic control and complications of type 1 diabetes in children and adolescents in Tanzania.	Cross-sectional study	All these children were treated with a conventional insulin regimen. The mean ± SD duration of diabetes was 4.76 ± 3.58 years. Only 1 child (1%) had good glycemic, 60 children (60.6%) had moderate glycemic control, 14 children (14.1%) had poor glycemic control, and 24 children (24.2%) had very poor glycemic control. At onset of diabetes, 75% of children presented with diabetic ketoacidosis (DKA); 89 children (89.80%) had at least one episode of DKA, and 55 children (55.67%) had symptomatic hypoglycemic episodes. Microalbuminuria was present in 29 (29.3%) and retinopathy in 22 (22.68%) children.

### Selection and characteristics of included studies

Together, the results of these articles provided definitions of T1D and prevalence rates. The internal (social realities) and external (foreign policies) contextual obstacles related to care delivery in SSA as well as the related strategies were also mentioned. In 26 articles, barriers related to T1D care delivery strategies in SSA were discussed; 9 articles did not focus on a specific disease but described barriers and strategies on chronic disease care delivery in general. Overall, 28 articles included information on T1D care delivery in SSA and 7 describe strategies for implementation. Some articles described both aspects of care delivery and strategies for implementing them (*n* = 3), although these varied according to socio-cultural and political contexts. Further information on the articles included in the qualitative synthesis is given in Table 1.

### Obstacles identified in care delivery of T1D in SSA

Table 3 summarizes the main obstacles in care delivery of T1D in SSA between 1990 and 2020 mentioned in the included articles. We distinguished three types of contextual obstacles: those related to the health structures of many SSA countries, to the patients themselves and to the socio-cultural context, to highlight the different levels at which the strategies are and can be focused. While some articles highlighted the need to address upstream, structural barriers (health structure, broader funding policies, sociocultural context) and patient and community engagement that can be supported independent of interactions with the health system, this review focused primarily on identifying patient and family barriers and priorities related to T1D care delivery.

**Table 3:** Obstacles in the care delivery of T1D in SSA

Levels	Obstacles identified
Health structure of Sub-Saharan African countries	- Education (schools) and prevention - Insufficient medical staff (lack of competence, learning culture and motivation) - Financial costs - Difficulties in accessing quality health structures (primary care) - Health policy oriented toward communicable diseases
Patients	- Poverty - Inability to follow a suitable diet - Compliance - Lack of formal and family support - Financial burden - Education (low health literacy)
Sociocultural	- Low social acceptance of the disease - Social representations and stigma - Difficulties in therapeutic adaptation - Access to information

The contextual obstacles mentioned in several articles can be grouped into three main levels:

1. *Health systems*,* services*,* and policies* [9, 24]. Many SSA countries have very limited resources which are notoriously insufficient to deal with the scale of the problem. Because policies and programmes develop and maintain momentum, decisions made in the early 2000s continue to affect the organization and delivery of care today. Despite the creation of specialized T1D care centers in some regions of SSA, the active role of these centers is limited due to the inequitable allocation of government resources and a lack of policies and strategies [42].
  The consequences of this resource insufficiency are grave. Doctors and nurses with specialized expertise in pediatric diabetes are rarely available or accessible. Families are forced to pay out of pocket for their child’s diabetes care and supplies since public health budgets as well as the public health system do not regularly provide such care [8]. High mortality and high complication rates occur in those who survive [1]. The most minimal care is beyond the means of many families who face additional costs, notably, consultations, travel, and indirect costs [8, 42, 47–49]. Secondary prevention (to prevent acute and chronic complications) and education are not promoted enough when they should be a priority [9]. The current organization and inadequate funding of T1D care do not support patient empowerment, knowledge, and capacity for self-care delivery [42]. As a result of these multiple access barriers, patients are forced to undertake long and costly travel to reach consultation centers with overworked doctors or nurses who are inadequately trained to manage T1D.
2. *Patients and families.* Three major obstacles at the level of patients and families often make adequate care delivery of the disease illusory: (1) The financial burden [6, 24]: As noted above, most of the minimal costs of adequate T1D care are not covered by public or charitable funding. In low- and middle-income countries it is impossible for many patients to ensure the cost of drugs, particularly insulin, and blood glucose monitoring supplies [42]. Relative to the very low incomes of most people in SSA, the cost of essential inputs and reagents such as insulin and glucose test strips for 4–10 tests per day is prohibitive. (2) Difficulty with adolescent adherence [17]: There is a lack of formal support [24] from families for people living with T1D to have an adequate coping environment and to have a daily assistant. (3) Lack of patient education [9, 45], specifically, the combination of inadequate basic education (subliteracy) and lack of diabetes-specific education (interpretation of blood sugar levels according to the many factors that can influence this, decision-making on insulin doses, carbohydrate count in relation to local food preferences and availability).
3. *Sociocultural context* [9, 29]. Because the notion of a chronic disease requiring lifelong treatment is often poorly accepted [9], when diabetes is well controlled, insulin treatment is sometimes interrupted [9] because of a belief that the diabetes has been cured. This can lead to dangerously high levels of blood sugars with high risk of development of an acute hyperglycemic crisis (diabetic ketoacidosis). Many patients do not consider usefully to carry out regular clinical and biological checks, which makes therapeutic adaptation uncertain, particularly insulin therapy. This can be explained by the financial burden, stigmatization, or diabetes distress and burnout. In SSA, weight loss (which in T1D occurs because of hyperglycemia), even if it is voluntary, arouses mistrust because it raises the possibility of HIV infection [29]. Ramadan fasting is another source of glycemic imbalance, especially in insulin-dependent diabetic patients [9]. Although the religion authorizes these patients not to fast, many patients with diabetes continue to fast for fear of judgment from neighbors or family. In SSA, the child with T1D not only remains surrounded by “mystery”, but is also the site of projected misrepresentations about the means of being affected, notably through a fear of contagion [9]. Traditional medicine retains an essential place in many African countries. Its practices can be dangerous when patients are advised to abandon effective treatments to resort to empirical drugs, but which have the advantage of being inexpensive and of being part of a pharmacopoeia integrated into the cultural past [9]. These challenges highlight the importance of access to education and information [24] for diabetic patients.

### Categorization of strategies implemented in the care delivery of T1D in SSA

The description of the obstacles related to care delivery of T1D in SSA highlights that the obstacles to care delivery of T1D exist at different levels. Several articles emphasized that general and context-specific barriers need to be assessed and analyzed to enable the development of effective strategies in care delivery of T1D in SSA [9, 24, 41]. In addition, the specific context of the patient population, their needs, their medical problems, and their social constraints must be recognized [40]. An overview of several barriers is provided in Table 2, but many other context-related barriers in the care delivery of T1D are conceivable and should be considered during a disease care delivery process in a structured way.

Strategies in the care delivery of T1D in SSA can be broadly classified as workflow-focused [15] (minimizing contextual barriers and create conditions to promote sustainable behavioral changes) or provider-focused [14, 19] (minimizing provider-level barriers and creating facilitators for adherence to disease-related recommendations). Studies identified a need for children and adolescents with T1D to receive more aggressive care and follow-up and also for more resources to be devoted to this non-communicable disease [39]. This requires functioning and patient-oriented health services with systems oriented towards ensuring good patient and population outcomes. Diseases such as T1D may represent a “marker” condition for effective health care systems, so that the care of patients with T1D could serve as a criterion against which the components of a fully functioning and effective health care system could be judged [15]. A system with such components, (including continuous drug supply, diagnostic facilities, training and retention of health workers, and patient education) is essential in the care delivery of other non-communicable diseases and chronic communicable diseases such as tuberculosis and HIV/AIDS [15]. In this way, attention to the requirements for specific conditions - in this case T1D - can also help strengthen health systems. While HIV and TB have been addressed in this way, T1D remains neglected [38].

Provider-focused strategies seek to minimize barriers at the provider level and create facilitators for adherence to disease-related recommendations. Limited healthcare resources should be focused on managing T1D and other risk factors to prevent complications [14]. This can be achieved using communication strategies to raise awareness of disease care delivery processes. The active participation of patients, families, the media, governmental and non-governmental organizations, and health workers can help overcome certain difficulties [40]. It is essential, as pointed out by Datye et al. [19] to “give providers the tools to assess and influence their patients’ barriers to adherence shows promise as a strategy that can be used universally by providers to improve adherence and, therefore, glycemic control in youth with T1D”.

### Elements of successful T1D care delivery strategies in T1D care delivery in SSA

**Fig. 2:**
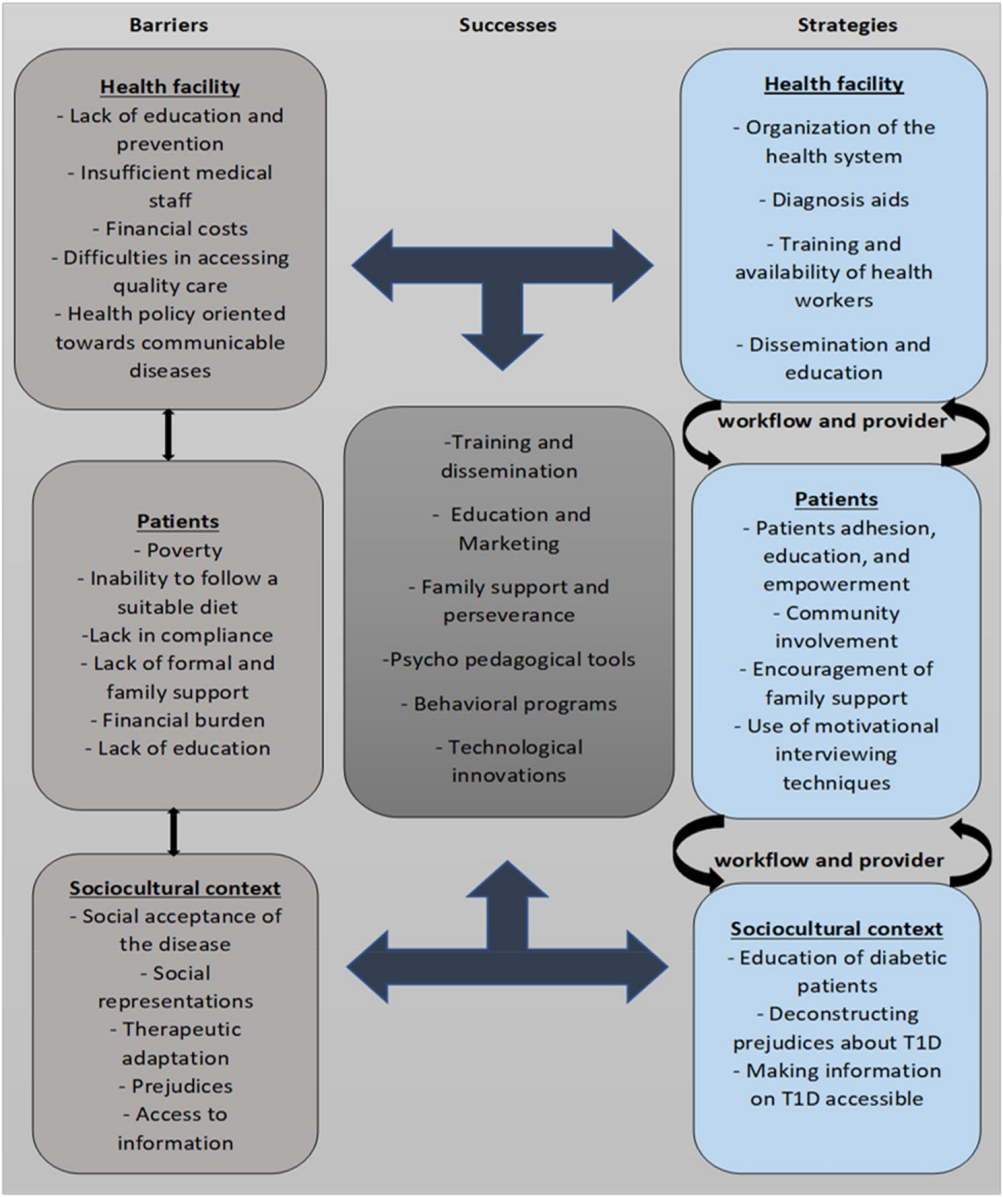
Structure of the different levels of obstacles, successful interventions and strategies highlighting the actors surrounding the care delivery of T1D in SSA

While strategies in the care delivery of T1D in SSA have been effective in modifying the knowledge and practice of health professionals, the existing evidence also indicates that good and structured care delivery of T1D can improve the health status of patients in SSA [9]. The review revealed the following aspects as core elements of successful strategies in the care delivery of T1D in SSA (see Fig. 2). Raising awareness of the costs of care can foster the review of national and international diabetes care (including T1D) policies and support programs, as well as dialogue and action by other key stakeholders, such as the industry [8]. Therefore, the provision of educational materials to the population (including written materials, didactic presentations, and interactive lectures) is essential to raise awareness and increase familiarity with disease care delivery methods and requirements. Continuous efforts in the education and training of health professionals and the population are necessary for rapid diagnosis and optimal care delivery of T1D [33, 41] (educational meetings and educational visits to raise awareness, audits and feedback, workshops, and interactive training sessions in small groups). Social interaction through family support is mentioned as a very relevant factor in the care delivery of T1D in SSA. Indeed, the significant interactions between T1D and important infectious diseases highlight the need and opportunity for health planners to develop integrated responses to communicable and non-communicable diseases [6]. This interaction may include educational outreach visits and marketing [45].

Effective interventions build on adolescents’ internal and external support (family, technology, and internal motivation) to simplify their diabetes care delivery and provide opportunities for adolescents to share the burden of care [17, 40]. Finally, common themes across successful interventions include perseverance, increased adolescent support (professional and family), and ongoing psychoeducational tools to motivate behavioral changes in daily life that promote adherence and reduce the daily burdens placed on adolescents [17]. These successful interventions include behavioral programs for patients and their family members as well as technological innovations, which aim to remind and inspire patients to follow their treatment regimens [17].

## Discussion

This scoping review provides an overview of the barriers identified in care delivery of children with T1D in SSA as well as a discussion of the components of the effective strategies identified. Few studies have been published since 2020, perhaps because of COVID-19 or the political and economic crises that have once again highlighted the difficulty of keeping DT1 in sight. In this review, we distinguished between contextual factors related to health care policies and services in SSA, patients themselves and socio-cultural contexts. The health policy of several Sub-Saharan African countries is strongly oriented towards the care delivery of communicable diseases, in particular HIV, to which most of the available funds are allocated with insufficient resources for the prevention and care of chronic conditions such as diabetes and hypertension [50]. In the early and mid 2000’s, some governments thought that these chronic non-communicable pathologies would not have time to appear because of the mortality due to HIV affecting young subjects [50]. Our review of perceived barriers and successful strategies in managing T1D in SSA responds to calls from experts to examine organizational resources (knowledge, attitudes, and beliefs), individual characteristics of healthcare providers, and the perception of actions and strategies in the care delivery of the disease [45]. It identifies shared core elements of successful strategies and puts them in the multilevel complex contexts of care delivery for this costly and under addressed condition. Despite the differences in the classification of obstacles, strategies in the care delivery of disease care delivery in SSA must take them all into account [9, 45].

Moreover, as Fassin mentions in Faire de la santé publique. Rennes, Edition ENSP, 2005, page 37, “*health problems are not only biological realities that specialists bring to light*,* but also epidemiological facts that they construct*” [51]. Care delivery of T1D in SSA today is also a cultural construction and an instrument of politics. In our review, the analysis of T1D outcomes according to cultural, political, and social contexts revealed new trends or manifestations in the care delivery of T1D in SSA. These include possible advances in care (combination screening), a loss of interest or new priorities in diabetes and considering local priorities (not to leave them out of the decision-making process since their health and that of their family is at stake). These complex interactions between bio-medical, social, psychological, cultural, political, and economic elements highlight that effective care delivery and/or prevention of T1D in SSA children must adopt multidisciplinary approaches [19, 20]. In addition, the review demonstrated that since the 1990s, different disease care delivery strategies in T1D in SSA related to barriers are being carried out. The predominance of retrospective and cross-sectional data supports the point of view of African researchers described by Ridde et al. [52]. These African researchers said that it is not easy to do prospective surveys due to low financial and material capacities for research [52].

The establishment of effective strategies in the care delivery of T1D in SSA includes important clinical components and requires changes in attitudes and behaviors of health professionals as well as some adaptation of the structural environment [45]. These clinical and structural changes require action in policy, funding, programme implementation, management, and evaluation. Although behavior can change even in the absence of changes in knowledge and attitudes, behavioral changes based on such changes are more permanent. All the obstacles described in this article, both internal and external to the Sub-Saharan context, can influence the knowledge, attitudes or behaviors of health professionals [53]. Health professionals in SSA must be aware of the guidelines on prevention, communication, legislation and have some knowledge of its content. Then, they must be equipped and supported to intervene by implementing effective strategies such as patient and family education. These strategies addressing the clinical and patient levels of the care delivery of T1D in SSA should focus on improving knowledge and also the opportunities, motivations, and capacities to act on this knowledge, as underlined by the Behaviour Change Wheel approach [54]. This requires multidisciplinary approaches and shared commitment from policy makers, managers, clinicians, families, and patients to improve the treatment of patients in SSA according to local contexts. Healthcare workers can effectively coordinate treatment, educate patients on self-care, and play an active role in secondary prevention [55]. For example, training programs such as those in Tanzania [56] or Mali [57] could be deployed to improve knowledge, with materials appropriately developed as guidelines and protocols. The International Insulin Foundation [58] has suggested a series of strategies to enhance T1D care delivery in SSA, including adherence to health guidelines. For such strategies to be developed and effectively implemented, barriers and common and contexts as well as those specific to a given context need to be assessed and removed. The effects of context on research and practice in the care delivery of T1D in SSA remain no less significant. Observation of disease management in the countries showed that many aspects of research dealing with T1D were being addressed. However, the research priorities of many of the published articles were generic barriers and strategies in care delivery, with little attention to how these cross-cutting issues play out in the specific case of T1D care delivery. Conversely, a second main observation concerns the independent and siloed actions of the different actors in the disease care delivery strategies in SSA. There is a need to coordinate actions, train, educate and build trust [59–62] in the contexts where actions are implemented for effective care delivery of T1D in SSA - and this must occur in the context of, and integrated with, both cross-cutting issues and contextual and technical specificities of many conditions and priorities. The treatment of T1D in low-income countries must consider the specific context of the patient population, their needs, their medical problems, and their social constraints [40, 63–66] - in relation to other levels and dimensions of care delivery, prevention, and promotion.

### Limitations

The main limitation of this review is that it included both the scientific literature and the grey literature to minimise the risk of missing relevant data sources. Although the inclusion of the grey literature broadened our scope, our analyses were nevertheless limited to the information provided in the data sources. It is possible that the information had additional components that were not identified. A more in-depth review would require identifying and analysing all the barriers and strategies put in place in terms of care delivery of T1D in SSA. A review of this nature requires many resources in terms of time and finances and was not possible in this case. Another limitation of the review is that it does not cover all territories of sub-Saharan Africa, which limits generalisability. That said, this work has identified barriers to implementing strategies that need to be addressed if sub-Saharan African countries are to improve the care of children with type 1 diabetes. Limiting our searches to studies in English and French is another limitation, but one that was unavoidable due to language proficiency. This would have allowed a broader consultation to help identify additional literature.

## Conclusion

The literature and issues focused on care delivery of children with T1D are not yet documented in all SSA countries. Based on a synthesis of evidence presented in 38 studies conducted in selected SSA countries, this scoping review described, categorized the most significant barriers, and identified effective strategies to implement the management of patients in SSA. The core elements of successful strategies for care delivery of T1D in SSA are patient self-management education, staff training, and social support (social interaction). Available evidence indicates that structured care delivery can improve the health of people living with T1D in SSA. Obstacles in the case and population care delivery of T1D and the health of people living with T1D need to be analyzed before strategies adapted to the context and specific target groups can be developed.

## Supplementary Information

Supplementary Material 1.

Supplementary Material 2.

## Data Availability

Not applicable.

## Abbreviations

PRISMA-ScR: Preferred Reporting Items for Systematic reviews and Meta-Analyses extension for Scoping Reviews
RCTs: Randomized Controlled Trials
SSA: Sub-Saharan African
T1D: Type 1 Diabetes
T2D: Type 2 Diabetes
UNICEF: The United Nations Children’s Fund
