# Supplementary material for: Barriers and strategies in care delivery for type 1 diabetes in Sub-Saharan Africa: a scoping review
Source: BMC Public Health. 2026 May 6;26:1968. doi: 10.1186/s12889-026-27354-9 (PMC13317225; doi:10.1186/s12889-026-27354-9)
Supplement: Supplementary file 2 — Supplementary Material 2. [file 12889_2026_27354_MOESM2_ESM.docx]

**Additional File 1. MEDLINE search strategy**

**Database:** Ovid MEDLINE(R) ALL <1946 to October 29, 2021>

| **Number** | **Search** | **Results from 30 Oct 2021** |
| --- | --- | --- |
| 1 | Diabetes Mellitus, Type 1/ | 79,810 |
| 2 | (diabet* or t1dm or t1d).ab,kf,kw,ti. | 697,576 |
| 3 | (diabet* adj4 type 1).ab,kf,kw,ti. | 54,569 |
| 4 | (t1dm or t1d).ab,kf,kw,ti. | 13,469 |
| 5 | 3 or 4 | 55,482 |
| 6 | 1 or 3 or 4 | 98,481 |
| 7 | exp "Africa South of the Sahara"/ | 230,693 |
| 8 | exp Patient Care Management/ | 851,298 |
| 9 | (management or care or support* or polic*).ab,kf,kw,ti. | 4,184,862 |
| 10 | 8 or 9 | 4,562,160 |
| 11 | 6 and 7 and 10 | 138 |
| 12 | 1 or 2 | 705,093 |
| 13 | 7 and 10 and 12 | 1,770 |
| 14 | 6 and 7 | 421 |
| 15 | limit 14 to yr="1990 - 2020" | 345 |

**Medline search strategy** (literature search performed: April 14, 2019)

1. Diabète infantile de type 1 /

2. Enfants /

3. Adolescents /

4. Service d’endocrinologie, hôpital /

5. Désordre psychologique /

6. Désordre alimentaire /

7. $ diabète, hw.

8. Complications /

9. Performance /

10. Prise en charge /

11. (Familles $, amis $, voisins ou support social $).tw.

12. ou / 1-11

13. (Thérapeutique ou thérapie$).tw.

14. Insuline /

15. Complications /

16. ou /12-15

17. Activité physique /

18. Pompe à insuline /

19. Régime alimentaire /

20. Hba1c /

21. Acidocétose diabétique /

22. Hypoglycémie /

21. Diabète néonatal /

22. (lois adj2 limitations) .tw.

23.  lj.fs.

23. OMS /

25. Fédération internationale du diabète /

25. Novo Nordisk /

27. (unité $, centre ou structure $).tw.

28. 23-25

29. Limite 28 à yr = 2010-2018

30. Limite 29 en anglais
